# Anti-endothelial cell antibodies in pathogenesis of vasculitis

**DOI:** 10.3389/fimmu.2025.1567293

**Published:** 2025-04-30

**Authors:** Tian Zhang, Liqun Li, Shengshi Huang, Maria N. Starodubtseva, Ju Liu

**Affiliations:** ^1^ Laboratory of Translational Medicine in Microvascular Regulation, Medical Research Center, The First Affiliated Hospital of Shandong First Medical University and Shandong Provincial Qianfoshan Hospital, Jinan, Shandong, China; ^2^ Shandong Provincial Key Medical and Health Laboratory of Translational Medicine in Microvascular Aging, The First Affiliated Hospital of Shandong First Medical University and Shandong Provincial Qianfoshan Hospital, Jinan, Shandong, China; ^3^ Laboraotry for Future Industry in Gene Editing in Vascular Endothelial Cells of Universities of Shandong Province, The First Affiliated Hospital of Shandong First Medical University and Shandong Provincial Qianfoshan Hospital, Jinan, Shandong, China; ^4^ Department of Clinical Laboratory, Qingdao Municipal Hospital, University of Health and Rehabilitation Sciences, Qingdao, Shandong, China; ^5^ Gomel State Medical University, Gomel, Belarus; ^6^ Institute of Radiobiology of NAS of Belarus, Gomel, Belarus

**Keywords:** vasculitis, endothelial cells, anti-endothelial cell antibodies (AECAs), angiogenesis, mechanical properties, stiffness

## Abstract

Vasculitis is a group of syndromes characterized by inflammation, presence of autoantibodies and endothelial cells (ECs) damage, which lead to stenosis or occlusion of the vascular lumen. Anti-endothelial cell antibodies (AECAs) are a heterogeneous group of autoantibodies in vasculitis. AECAs bind to antigens and membrane-bound proteins of ECs, inducing inflammation, coagulation, and apoptosis. In this review, we discuss the pathological role of AECAs in different types of vasculitis. In addition, AECAs potentially induce alterations of ECs mechanical properties, and subsequently promotes angiogenic phenotypes in the occurrence of vasculitis.

## Introduction

Vasculitis encompasses a spectrum of immune-mediated diseases marked by the immune system’s aberrant attack on blood vessels in various body organs, such as the skin, lungs, and kidneys. This disorder is categorized based on the size of the affected blood vessel (1). In 2012, the Chapel Hill International Consensus Conferences established and standardized vasculitis nomenclature. The classification of vasculitis includes seven primary categories: large vessel vasculitis, medium vessel vasculitis, small vessel vasculitis, variable vessel vasculitis, single-organ vasculitis, vasculitis associated with systemic diseases, and vasculitis linked to probable etiology (2) (**Table 1**).

**Table 1:** The classification of vasculitis.

Classification		Diseases
LVV		TAK, GCA
MVV		PAN, KD
SVV	AAV	MPA, GPA, EGPA
	Immune complex SVV	IgAV, anti-glomerular basement membrane disease, cryoglobulin vasculitis
VVV		BD, Cogan’s syndrome
SOV	Diffuse SOV	Cutaneous polyarteritis nodosa, Cutaneous leukocytoclastic angiitis, Primary angiitis of the central-nervous system, Lower-limb-restricted polyarteritis nodosa
	Focal SOV	Localized vasculitis of the aorta, Vasculitis of the gastrointestinal tract, urogenital tract, breast, Retinal vasculitis, Muscular vasculitis
VASD		SLE, Sarcoidosis
VAPE		HCV-associated cryoglobulinemic vasculitis, HBV-associated vasculitis, Syphilis-associated aortitis, Drug-associated immune complex vasculitis, Drug-associated ANCA-associated vasculitis

This table summarizes the classification of vasculitis based on CHCC in 2012. LVV, large vessel vasculitis; GCA, giant cell arteritis; TAK, Takayasu’s arteritis; MVV, medium vessel vasculitis; KD, kawasaki disease; SVV, small vessel vasculitis; AAV, anti-neutrophil cytoplasmic antibodies (ANCA)-associated vasculitis; MPA, microscopic polyangiitis; GPA, granulomatosis with polyangiiti; IgAV, IgA vasculitis (Henoch-Schönlein); VASD, vasculitis associated with systemic disease; VAPE, vasculitis associated with probable etiology; VVV, variable vessel vasculitis; BD, Behçcet’s disease; SLE, systemic lupus erythematosus; HCV, hepatitis C virus; SOV, single-organ vasculitis.

The occurrence of vasculitis typically precipitates vascular stenosis or occlusion, which in turn induces organ ischemia or contributes to the attenuation of blood vessel walls, thereby facilitating the formation of aneurysms or provoking hemorrhage. Central to this pathophysiological process is ECs injury, accompanied by the infiltration of leukocytes, which collectively underpin the common pathological mechanism inherent in such conditions (3). Contemporary research emphasizes the critical role of autoantibodies in the etiology of vasculitis. These autoantibodies in vasculitis predominantly target elements of the immune system or act directly against ECs (4–6). Anti-Endothelial Cell Antibodies (AECAs) are identified as one of the key autoantibodies in the context of vasculitis. These AECAs are often detected in patients diagnosed with vasculitis syndrome and are considered to be reliable indicators of both the activity and severity of the condition, as evidenced by various studies (7–10). AECAs have been identified in a spectrum of vasculitis disorders, encompassing Takayasu arteritis (TAK) (11), giant cell arteritis (GCA) (12), Kawasaki disease (KD) (13), granulomatosis with polyangiitis (GPA) (14), microscopic polyangiitis (MPA) (15), IgA vasculitis (IgAV) (16), Behçet’s disease (BD) (17), systemic lupus erythematosus (SLE) (18), and sarcoidosis (19). The presence of AECAs in vasculitis highlights their potential role in the pathogenesis and clinical progression of vasculitis. The pathological mechanisms include activation of ECs, induction of coagulation and apoptosis. The above conditions in turn lead to angiogenesis and changes in the mechanical properties of ECs. In this review, we summarized our current understanding of the diverse mechanisms of AECAs in ECs dysfunction, highlighting the angiogenesis and cellular mechanics properties of AECAs in vasculitis.

## AECAs characteristics

### Antigen types

AECAs are circulating antibodies that recognize various antigenic determinants on ECs (20). Most scholars believe that AECAs’ antigens are classified into three categories: “planted” antigens, constitutively expressed antigens, and cryptic antigens (21, 22).

“Planted” antigens adhere to the endothelium either directly, such as myeloperoxidase (MPO) and β2-glycoprotein I (β2-GPI), or indirectly adhere through DNA or DNA-histone complexes. Because of the presence of “planted” antigens, AECAs are capable cross-link with many autoantibodies (23). Constitutively expressed antigens include human leukocyte antigen (HLA) I antigens, cardiolipin, and other phospholipid components, as well as extracellular matrix components (24). HLA I antigens present on ECs represent some of the antigenic epitopes for AECAs. Cardiolipin and other phospholipid structures are some of the antigenic determinants for a subset of AECAs in vasculitis. In addition, different extracellular matrix components such as collagen type II, IV, VII, vimentin, tropomyosin, and laminin are also target antigens for AECAs (25). Cryptic antigens include HLA II antigen and proteinase 3 (PR3). HLA II and PR3 determinants are present only on activated ECs and represent target antigens for AECAs (26). Cytokine activation human umbilical vein endothelial cells (HUVECs) lead to PR3 translocation from the cytoplasm to the cell membrane. This is followed by the development of vasculitis in conjunction with AECAs.

Other antigens include α-enolase, heat shock protein (HSP) 60, HSP70, ribosomal P protein P0, calreticulin, and tubulin (27–29). Besides, adhesion molecules such as intercellular cell adhesion molecule-1 (ICAM-1) also serve as specific target antigens for AECAs (29) (**Table 2**). Numerous target antigens for AECAs in vasculitis have been studied, including HLA in Takayasu’s arteritis and KD (30, 31), β2-GPI in GCA (32) and IgAV (33), DNA, PR3, MPO and phospholipid in anti-neutrophil cytoplasmic antibodies-associated vasculitis (34), HSP60, HSP70 in GCA and KD (35). However, the specific pathogenesis has not been elucidated (**Table 2**).

**Table 2:** The classification of AECAs target antigens.

Classification	Antigens	
"Planted"antigens	MPO	
	B2-GPI	
	DNA or DNA histone complexes	
Constitutively expressed antigens	HLA I antigens	
	Cardiolipin and other phospholipids components	
	Extracellular matrix components	collagen type II, IV, VI
		vimentin
		tropomyosin
		laminin
Cryptic antigens	HLA II antigen	
	PR3	
Other antigens	α-enolase	
	HSP60, HSP70	
	ribosomal P protein P0	
	CRT	
	tubulin	

This table summarizes the classification of AECAs target antigens. MPO, myeloperoxidase; β2-GPI, β2-glycoprotein I; HLA, human leukocyte; PR3, protease 3; HSP, heat shock protein; CRT, calreticulin.

However, it is crucial to emphasize that AECAs exhibit cross-reactivity with multiple cell types, as their epitopes are expressed not only on ECs but also on fibroblasts, leukocytes, and monocytes (36). Although ECs models are commonly used in AECA studies, their biological properties differ from pathological targets. For example, HUVECs derive from embryonic umbilical veins. These cells exhibit distinct phenotypic features, including short-chain von Willebrand factor (vWF) multimers, compared to adult arterial or capillary ECs. This model-target mismatch causes discrepancies between experimental and clinical findings. Vascular bed heterogeneity influences AECAs targeting specificity. In sarcoidosis, AECAs react more strongly with bone marrow ECs than HUVECs. In BD, they show higher reactivity with omental microvascular ECs versus HUVECs. Conversely, in TAK, AECAs activate HUVECs but not microvascular ECs (23, 37). Additionally, epigenetic modifications (e.g., DNA methylation) in ECs show significant heterogeneity, potentially influencing antigen accessibility and immunogenicity. Furthermore, AECAs detection has limited diagnostic value in clinical practice. These antibodies lack a specific target antigen to guarantee immune specificity. Current detection methods show suboptimal accuracy and face technical challenges in standardization (38).

### Pro-inflammatory effects

**Figure 1:**
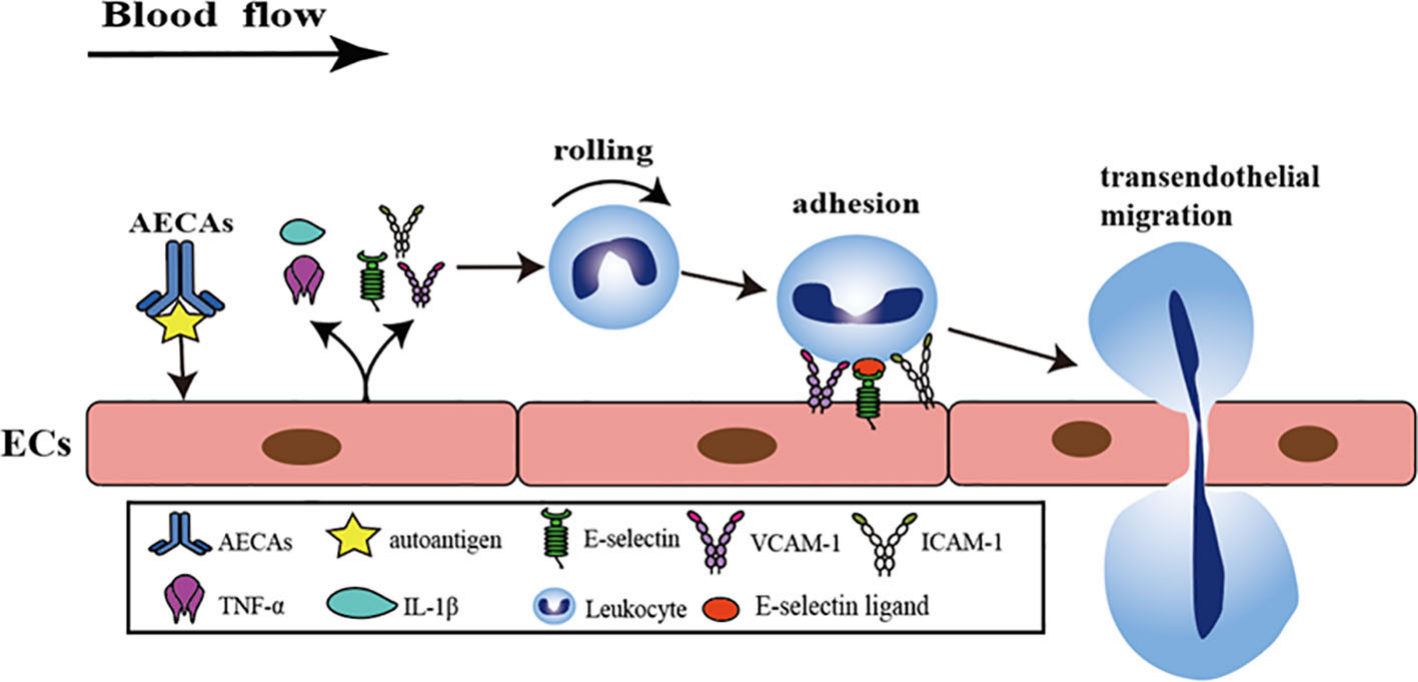
AECAs pro-inflammatory effects in ECs. AECAs bind to antigens on the surface of ECs and release IL-1β, TNF-α, E-selectin, ICAM-1, and VCAM-1 to promote leukocyte rolling. ICAM-1, VCAM-1, and E-selectin bind to leukocytes via ligands to promote adhesion and eventual leukocyte crossing of the endothelial gap (AECAs, anti-endothelial cell antibodies; ICAM-1, intercellular cell adhesion molecule-1; VCAM-1, vascular cell adhesion molecule-1; IL-1β, Interleukin-1β; TNF-α, tumor necrosis factor-α).

**Figure 2:**
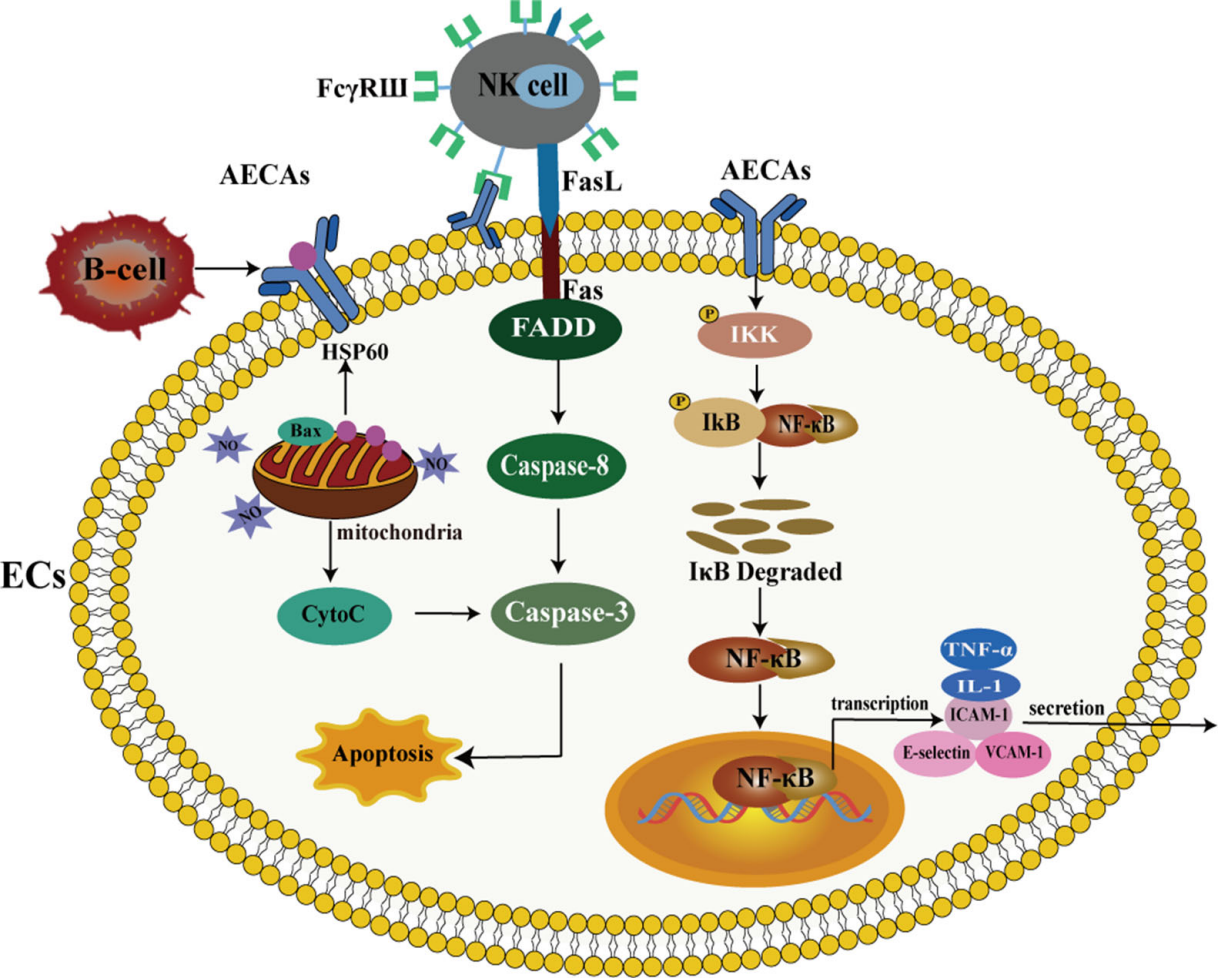
AECAs pro-apoptotic and pro-inflammatory effects in ECs. On the left, AECAs regulate mitochondrial pathway-mediated apoptosis through NO and HSP60 production. When ECs are injured, HSP60 is located on the plasma membrane and separated from Bax. With the decrease of HSP60, Bax moves from the cytoplasm to the mitochondria and is accompanied by the release of Cytoc, which activates caspase-3 and induces apoptosis in ECs. FcγRШ mediates NK cells, binds to AECAs and directly kills antibody-linked target cells. AECAs causes ECs apoptosis through Fas/FasL interaction. Upon binding of Fas to its cognate ligand FasL, Fas is recruited in the cytoplasm via the death domain of the intracellular FADD and caspase-8-associated Fas, forming a death-inducing signaling complex consisting of Fas, FADD and caspase-8, which activates caspase-3 and ultimately leads to apoptosis. On the right, AECAs interact with ECs antigens and activate IKK. Activated IKK phosphorylates IκB protein and induces ubiquitination and degradation of IκB, which subsequently leads to NF-κB activation. After NF-κB translocation into the nucleus, the expression of IL-1, TNF-α, ICAM-1 and VCAM-1 is upregulated (AECAs, anti-endothelial cell antibodies; ECs, endothelial cells; NO, nitric oxide; HSP60, heat shock protein 60; NK, Natural Killer; IκB, inhibitor of NF-κB; IKK, IκB Kinase; Fas, factor associated suicide; FasL, Fas ligand; FADD, Fas-associating protein with a novel death domain; IL-1, Interleukin-1; TNF-α, tumor necrosis factor-α; ICAM-1, intercellular cell adhesion molecule-1; VCAM-1, vascular cell adhesion molecule-1).

Under normal conditions, leukocytes race in laminar blood and do not adhere to ECs (39). As inflammation progresses, ECs produce tumor necrosis factor-α (TNF-α), interleukin (IL)-1, IL-6, enhance the expression of ICAM-1, vascular cell adhesion molecule 1 (VCAM-1), and synthesize E-selectin, causing leukocytes to roll on ECs (40–43). E-selectin binds to leukocytes through their ligands and promotes leukocyte adhesion (44). There is evidence that AECAs bind to β2-GPI on the surface of ECs, up-regulating IL-1, TNF-α, ICAM-1, VCAM-1 and E-selectin, and increasing leukocyte adhesion to ECs (45–47). Leukocyte adhesion leads to endothelial dysfunction, decreased endothelium-dependent vasodilation, excessive capillary filtration, and increased protein leakage in venules (48) (**Figure 1**). The increased expression of AECAs mediates ECs injury and activates the nuclear factor-κB (NF-κB) signaling pathway (49). NF-κB activation occurs through the degradation of the inhibitor of NF-κB (IκB). The degradation of IκB requires the activation of IκB kinase (IKK) to phosphorylate IκB. IKK increases IκB degradation, leading to nuclear translocation of NF-κB subunit and NF-κB mediated expression of pro-inflammatory cytokines (50). Leukocyte adhesion is further promoted by up-regulating the expression of IL-1, TNF-α, ICAM-1, VCAM-1, and E-selectin (51–53) (**Figures 1**, **2**).

### Pro-coagulant effects

**Figure 3:**
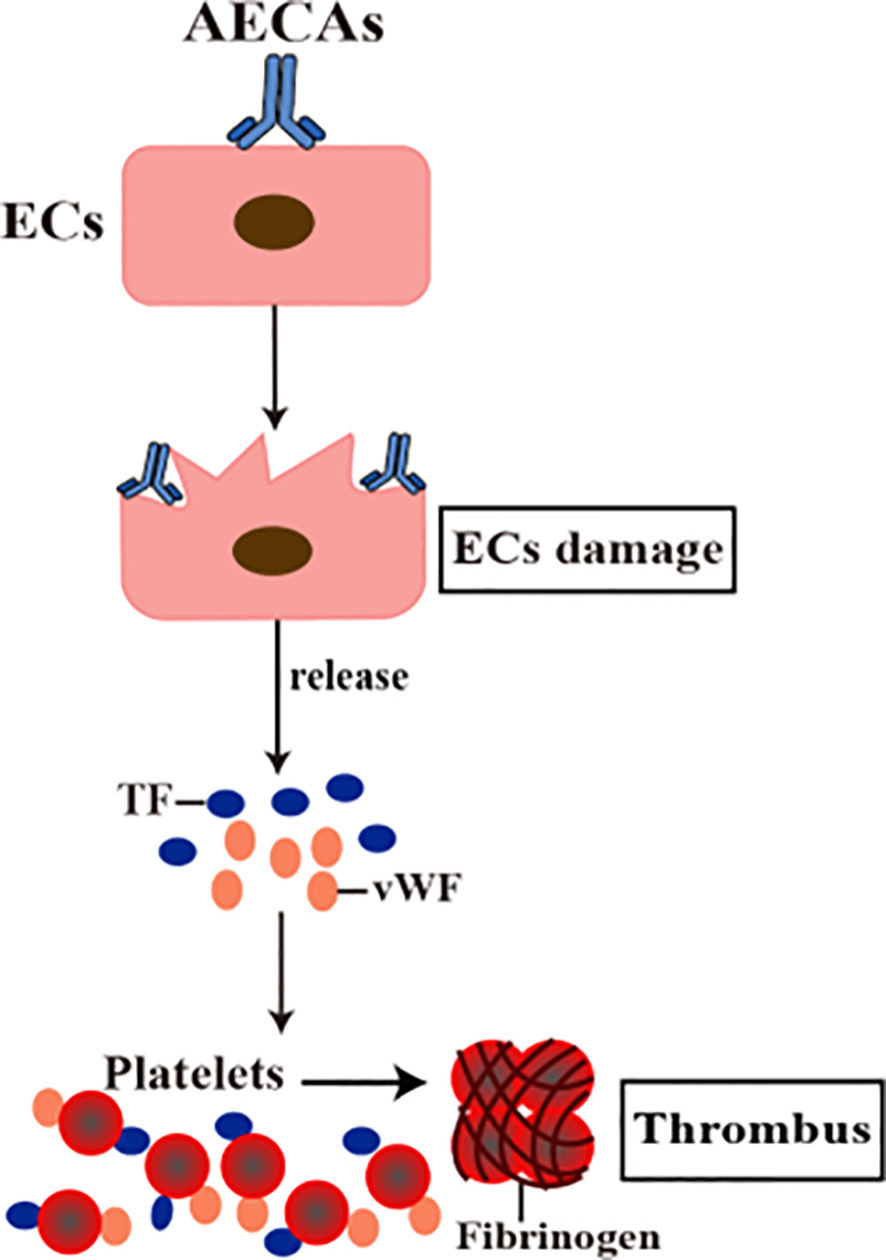
AECAs pro-coagulant effects in ECs. AECAs act on ECs, resulting in damage to ECs and release of coagulation factors such as vWF and TF. Platelets are activated by vWF and TF. Thrombus formation with vWF, TF and fibrinogen in damaged ECs (AECAs, anti-endothelial cell antibodies; ECs, endothelial cells; vWF, Von Willebrand factor; TF, tissue factor).

ECs have the function of maintaining blood flow and regulating blood coagulation (54). When ECs are damaged, coagulation becomes dysfunctional, leading to occlusive thrombosis, local tissue and organ necrosis (55). vWF and tissue factor (TF) are essential indicators of thrombosis caused by ECs injury (56–58). Evidence suggests that AECAs stimulate ECs to secrete vWF and its ultra-large polymers (ULvWF) (13, 56). ULvWF located on the surface of damaged ECs interacts with platelets (59). Platelets are activated by vWF and form thrombus with vWF and fibrin in damaged ECs. Activated platelets release P-selectin and promote the release of neutrophil extracellular trap (NET), comprising condensed chromatin and histone. Histone induce platelet aggregation and platelet accumulation (60, 61). In addition, the interaction between AECAs and ECs surface antigen leads to the production of tissue factor (TF), and TF activity is dose-dependent on the AECAs titer, contributing to thrombosis (13, 56, 62). In summary, AECAs binding to ECs promotes the release of vWF, ULvWF, and TF, causing inflammation and thrombosis (**Figure 3**).

### Pro-apoptotic effects

At present, there are two ways to induce apoptosis: the mitochondrial pathway, also known as the endogenous pathway, activates caspase by regulating mitochondrial membrane permeability and releasing apoptosis-activating factors (63). The death receptor-mediated pathway activates caspase by combining extracellular death receptors and corresponding ligands (64, 65). Both pathways are characterized by caspase activation. The following evidence provides that how AECAs induce ECs apoptosis through two pathways (66) (**Figure 2**).

### Mitochondrial pathway

Mitochondrial proteins such as heat shock protein 60 (HSP60) and nitric oxide (NO) mediate ECs apoptosis. HSP60 exists in mitochondria. 15% to 20% of HSP60 exists in the cytoplasm and forms a complex with Bax in the cytoplasm (67). When ECs are stressed or damaged, HSP60 is located on the plasma membrane and separated from Bax. With the decrease of HSP60, Bax moves from the cytoplasm to mitochondria and is accompanied by the release of cytochrome c, and apoptosis is triggered. As a result, apoptosis is initiated, HSP60 is released, and this, in turn, accelerates the activation of procaspase-3 (67). The abnormal expression of HSP60 on the cytoplasmic membrane makes ECs susceptible to apoptosis (68). As a result, HSP60 dissociates from the plasma membrane and interacts with AECAs. Bax moves to mitochondria, leading to the release of cytochrome c and further activation of caspases-3, triggering apoptosis (69). Furthermore, ECs undergo apoptosis via the mitochondria-dependent pathway regulated by NO production (70). There is evidence that AECAs induce ECs apoptosis through a NO-mediated mechanism in dengue virus infection (66). NO-regulated ECs injury thus plays a role in disrupting ECs integrity and contributing to the pathogenesis of vasculopathy induced by AECAs. In conclusion, AECAs exert apoptosis by acting on ECs through the mitochondrial pathway via mitochondrial-associated proteins such as HSP60 and NO.

### Fas/FasL pathway

AECAs contribute to the apoptosis of ECs through antibody-dependent cell-mediated cytotoxicity (ADCC), underscoring their role in the pathophysiological mechanisms affecting vascular integrity and function (71). Natural Killer (NK) cells are the mediators of ADCC. FcγRIII mediates on NK cells, binding to AECAs (72), and directly kills the target cells attached to the antibody. NK cells have two different cytotoxic mechanisms. The first is granule-mediated apoptosis, which depends on the synergistic effect of the perforin and serine protease granzymes (73). The other is factor-associated suicide (Fas)/Fas ligand (FasL) interaction mediated apoptosis (74). Existing research indicates that the granzyme A gene is expressed in systemic sclerosis, suggesting that granzyme mediates ECs injury (71), but whether AECAs are induced by granzyme A expression has not been functionally confirmed. A study has confirmed that AECAs induce apoptosis of human dermal microvascular ECs through Fas, while blocking anti-FasL antibodies inhibited ECs apoptosis (75). Therefore, AECAs may cause apoptosis of ECs through the Fas/FasL interaction. After Fas binds to its homologous ligand FasL, Fas recruits Fas-associated death domain (FADD) and caspase-8/10 in the cytoplasm through the death domain of the intracellular segment, forming a death-inducing signal complex composed of Fas, FADD and caspase-8/10, which activates caspases-3 and eventually leads to apoptosis (76, 77).

## Pathological role of AECAs in vasculitis

In examining the pathological role of AECAs in vasculitis, it is imperative to acknowledge their pervasive presence across a multitude of vasculitic disorders. The function of AECAs extends beyond serving merely as biomarkers of disease; they play a pivotal role in the pathophysiology of disease development and progression (12, 78). We will delve into the specific roles of AECAs in various forms of vasculitis, as well as their impact on clinical manifestations of these diseases (as shown in **Table 3**).

**Table 3:** The pathological role of AECAs in vasculitis.

Diseases	Pathological role
TAK	VCAM-1, ICAM-1, E-selectin, IL-4, IL-6, IL-8↑; NF-κB activation
GCA	–
KD	E-selectin, VCAM-1, ICAM-1, TNF-α, IL-6↑; NF-κB activation
GPA	MICA, VAP-1↑; SAPK/JNK, NF-κB activation
MPA	–
IgAV	ERK-1 phosphorylation, IL-8↑
BD	ICAM-1, ERK1, ERK2↑
Sarcoidosis	Fas/FasL signaling pathway
SLE	sE-selectin, sVCAM-1, endothelin-1↑

This table summarizes the vasculitis associated with AECAs as well as the pathological roles. TAK, Takayasu’s arteritis; GCA, giant cell arteritis; KD, kawasaki disease; GPA, granulomatosis with polyangiiti; MPA, microscopic polyangiitis; IgAV, IgA vasculitis; BD, Behçcet’s disease; SLE, systemic lupus erythematosus; ICAM-1, intercellular cell adhesion molecule-1; VCAM-1, vascular cell adhesion molecule-1; IL-4, Interleukin-4; IL-6, Interleukin-6; IL-8, Interleukin-8; TNF-α, tumor necrosis factor-α; MICA, MHC class I-related antigen A; VAP-1, vascular adhesion protein-1; SAPK/JNK, stress-activated protein kinase/c-Jun N-terminal kinase; ERK, extracellular signal-regulated kinases; Fas, factor associated suicide; FasL, Fas ligand.

### Large vessel vasculitis

TAK is a chronic idiopathic granulomatous large-vessel vasculitis that affects the aorta, its main branches, and pulmonary arteries (79). Evidence has shown that AECAs are related to the pathogenesis of TAK (78). The positive rates of IgG and IgM AECAs in TAK patients were over 68%, and the titer of IgM AECA was higher (80). Study shows that most patients with TAK have circulating AECAs, which are directed predominantly to a triplet of aortic ECs antigens ranging in size from 60kD to 65kD. These AECAs induce the expression of VCAM-1 and E-selectin, as well as the production of inflammatory cytokines such as IL-4, IL-6, and IL-8 by aortic ECs, leading to the apoptosis of aortic ECs (81). Another study found that AECAs derived from a single patient with TAK were found to activate HUVEC, as shown by increased secretion of IL-6, vWF and increased expression of VCAM-1, ICAM-1, E-selectin, associated with NF-κB activation and increased adhesion of monocytes to these cells (82).

GCA, also known as temporal arteritis, is a primary systemic vasculitis involving large and medium vessels. However, with the advancement of vascular imaging technology, GCA is now considered to be a systemic disease beyond the superficial temporal artery, which causes large artery stenosis or aortic involvement (aortitis, aneurysm formation, or dissection) (83–86). A study has confirmed that IgG, IgM, and IgA AECA are positive in GCA patients, with IgA AECA is highly expressed, and IgG AECA plays a vital role in maintaining homeostasis (12).

### Medium vessel vasculitis

KD is the most representative disease of medium vessel vasculitis. The research indicates that AECAs are related to KD pathogenesis and clinical diagnosis. E. Grunebaum (87) et al. confirmed that AECAs activated ECs, thereby increasing the secretion of IL-6, the expression of adhesion molecules such as E-selectin, VCAM-1, ICAM-1, and the adhesion of U937 cells to HUVECs. A study has found that the high expression of E-selectin in acute KD is related to the activation of NF-κB in vascular ECs and then increase the release of TNF-α (88). In conclusion, AECAs activate ECs through the NF-κB signaling pathway and increase the expression of cytokines such as IL-6, TNF-α and adhesion factors such as VCAM-1, ICAM-1, E-selectin, leading to impaired function of ECs, and participating in the pathogenesis of KD.

### Small vessel vasculitis

GPA was formerly known as Wegener’s granuloma, and is characterized by necrotizing vasculitis of small vessels and granulomatous inflammation (89). Current research has confirmed that AECAs are a critical factor in the pathogenesis of GPA. Many patients with GPA have AECAs that react with human kidney microvascular ECs. Stimulation of human kidney microvascular ECs with IgG AECA upregulated MHC class I - related antigen A (MICA) and vascular adhesion protein-1 (VAP-1) expression, triggered rapid Ca^2+^ flux, induced stress-activated protein kinase (SAPK)/c-Jun N-terminal kinase (JNK), specific phosphorylation of transcription factor c-Jun and activating transcription factor-2, and activated NF-κB. However, specific SAPK/JNK inhibitors significantly reduced AECAs-induced chemokine production and phosphorylation of c-Jun and activating transcription factor-2 and eliminated MICA protein expression (14). Taken together, the elevated expression of AECAs mediates the pathogenesis of GPA and is associated with the SAPK/JNK pathway and the endothelial inflammatory protein VAP-1.

MPA is a systemic autoimmune necrotizing vasculitis. A reported study showed the presence of AECAs in patients with MPA and suggested a correlation between AECAs titer and disease activity (15). Régent et al. confirmed that purified serum IgG from patients with MPA induced extracellular regulated kinase (ERK) phosphorylation in ECs than IgG from healthy controls *in vitro*, which supports a possible pathogenic role of AECAs in MPA (90).

IgAV, also known as Henoch-Schönlein. IgAV is a type of vasculitis with IgA1-dominated immune deposits affecting small vessels (capillaries, veins or arterioles) (91). Studies found that AECAs are involved in the pathogenesis of IgAV. Yang YH (92)and Cynthia C (16)et al showed that AECAs were significantly elevated in patients with acute Henoch-Schönlein (16, 92). The mechanism of occurrence is related to the binding of AECAs to EC antigen, leading to ERK-1 phosphorylation, which activates protein-1 phosphorylation and promotes IL-8 expression (93).

### Variable vessel vasculitis

BD is a variable-vessel vasculitis with a predominance of recurrent thrombophlebitis, thrombosis and cutaneous vasculitis (94). Direskeneli H (95) found a high rate of positive AECAs in patients with BD. AECAs are associated with disease activity in BD. Studies have found that AECAs positive sera from BD patients lead to changes in the expression of adhesion molecules on the cell surface of human dermal microvascular ECs (HDMECs) and promote the adherence of T lymphocytes to HDMECs and, thereby initiating or amplifying inflammatory vascular injury (96). IgM AECAs play a pathogenetic role in BD by activating ECs directly. In addition, extracellular signal-regulated kinases (ERK) 1 and 2 were involved in the expression of ICAM-1 on HDMECs stimulated by AECAs (97, 98). Although the exact pathogenesis of BD is still unknown, it seems that it involves at least three steps: activation of ECs, adhesion molecule expression, and lymphocyte adhesion. Activation of ECs facilitates leukocyte traffic and thus initiate an inflammatory injury.

### Vasculitis associated with systemic disease

Sarcoidosis is an inflammatory disease characterized by granuloma (abnormal inflammatory cell mass) in almost all organs that usually affects the lungs, lymph nodes, skin, and eyes (99). Naoki Inui et al. showed that AECAs positive rate and AECAs level in sarcoidosis patients’ serum and bronchoalveolar lavage fluid were significantly increased (20). Initial AECAs levels were further elevated in patients with multiple organ involvement or requiring glucocorticoid therapy. AECAs leading to ECs apoptosis were found to be mediating the pathogenesis of sarcoidosis (100). The mechanism induced by ADCC via Fas/FasL interactions (75). Taken together, AECAs induces ECs apoptosis through the Fas/FasL pathway, which is one of the pathological mechanisms in the development of sarcoid vasculitis.

SLE is a chronic autoimmune disease characterized by a multisystem disorder caused by immune dysregulation (101). Study confirms that AECAs are useful diagnostic and prognostic tools for SLE patients (102, 103). The expression of AECAs is increased at the initial stage of vascular injury in SLE. In SLE, AECAs demonstrate significant correlations with elevated serum concentrations of sE-selectin and sVCAM-1, mechanistically contributing to ECs activation through enhanced leukocyte adhesion and pro-inflammatory signaling (46, 104, 105). Notably, ribosomal P0 protein has been identified as an autoantigen recognized by AECAs, with its immunoreactivity showing specific association with SLE disease manifestations (106). Experimental evidence indicates that IgM-class AECAs co-localizing with immune complexes induce endothelial endothelin-1 overexpression, mediating the initiation and progression of microvascular injury in SLE patients (107). Furthermore, anti-phospholipid antibodies and AECAs exhibit functional cross-reactivity. Specific anti-annexin V antibodies bind to membrane-associated epitopes, inducing phosphatidylserine exposure and activating apoptotic pathways in ECs. These mechanisms are implicated in SLE-associated vascular pathology (29, 108).

## Pathogenic mechanisms of AECAs in vasculitis

### Angiogenesis

Angiogenesis in vasculitis is related to inflammatory factors secreted by ECs. Research has shown that in GCA, when a large amount of carriers of free hemoglobin with angiogenic properties are produced, the levels of TNF-α and IL-6 were increased. In GCA vasculopathy, leukocyte constitutive (PECAM-1, ICAM-1, ICAM-2, and P-selectin) and inducible (E-selectin and VCAM-1) ECs adhesion molecules are overexpressed by ECs of adventitial micro-vessels and neo-vessels (109–111). The interactions of leukocytes with these ligands are responsible for forming inflammatory infiltrates in GCA lesions (112). GCA is characterized by the release of pro-inflammatory cytokines such as IL-1, TNF-α, and IL-6 during the acute systemic phase (113), which influence vascular responses such as vessel occlusion or regeneration and participate in the pathogenesis of GCA inflammatory lesions (114). In KD, ECs of newly formed vessels in coronary aneurysms express E-selectinandVCAM-1, which is involved in leukocyte adhesion to ECs (115). In contrast, luminal ECs of coronary arteries without aneurysms do not express E-selectin and VCAM-1, such as ECs of newly formed vessels in polyarteritis nodosa and GCA (112). In conclusion, angiogenesis is involved in the occurrence and development of vasculitis. Newly formed blood vessels express leukocyte adhesion molecules, such as VCAM-1, ICAM-1, and E-selectin, providing a new location for leukocytes to invade the blood vessel wall (112). In addition, new vessels expand the surface of ECs, providing an additional source of cytokines and chemokines that amplify the inflammatory process (116).

Studies have shown that AECAs induce the activation of ECs and secretion of inflammatory cytokines such as IL-1β and TNF-α, coagulation factors such as vWF, and adhesion molecules such as ICAM-1, VCAM-1, E-selectin, leading to vascular inflammation and occlusion, and participating in the occurrence of vasculitis (117, 118). After AECAs induce vascular damage, minimal proliferation, fibrosis, and thrombosis lead to narrowing or occlusion of the vascular lumen, causing tissue hypoxia and ischemia. The hypoxia-ischemic environment resulting from vascular lumen stenosis or occlusion is a powerful signal for new angiogenesis, ultimately leading to angiogenesis (119).

### Mechanical properties

Mechanical properties are significant for the normal functioning of ECs. They determine the integrity and mechanical stress resistance of an ЕС monolayer and regulate the functions of the endothelium under constant mechanical load from the side of the blood flow. Cells are known to be viscoelastic materials, and the origin of their mechanical properties is determined by the cytoskeleton (120). The cytoskeleton is a multi-hierarchical network structure within the cell with protein fibers as the main component, consisting of three types of protein fibers: actin filaments, tubulin microtubules, and a group of polymers known collectively as intermediate filaments. The cytoskeleton has several broad functions: it spatially organizes cellular contents; it connects the cell physically and biochemically to the external environment; it is involved in intracellular and extracellular transport and signal transduction; it generates coordinated forces that enable cells to move and change shape (121).

Several works reported cytoskeletal remodeling in response to the ligation of AECAs to ECs (122–125). Stimulation of ECs with HLA class I was revealed to activate stress fiber formation via a mechanism that did not include any detectable change in intracellular Ca^2+^ concentration, but induced Myosin light-chain phosphorylation and stress fiber assembly involving myosin light-chain (MLC) kinase and Rho-kinase (ROCK) in an ERK1/2-dependent manner. Molecular aggregation of HLA class I molecules with antibodies leads to the recruitment of integrin ß4 and the subsequent activation of intracellular signals that increase Rho-GTP activity, induce phosphorylation of ROCK, and trigger the assembly and phosphorylation of focal adhesion kinase, Src and paxillin at the focal adhesions to stimulate actin reorganization (124). The ligation of HLA class II to ECs was shown to induce necrotic cell death via a mechanism of lysosomal membrane permeabilization involving the reorganization of the actin cytoskeleton and the formation of actin stress fibers. The effect was downregulated by the actin polymerization inhibitor cytochalasin D and inhibition of Rho GTPases (125). More early work revealed that autoantibodies from a subset of advanced type 2 diabetes activate ROCK, and induce stress fiber formation and apoptosis in ECs (122). Changes in the stiffness of ECs occur in vascular inflammation (126). The stress fiber formation can increase the EC stiffness by a factor of 2-10 (127).

The mechanical properties of ECs depend also on the mechanical properties of their environment. An increase in substrate rigidity correlates with an overall increase in EC stiffness and apparent viscosity that is associated with the reorganization of actin cytoskeleton. Conversely, the cells on the soft substrate were more deformable and less viscous that is related to actin disordering (128). Inflammation, as a pathological factor of vasculitis, leads to increased vascular stiffness, and vascular stiffness is correlated with the degree of inflammation (129, 130). Inflammatory mediators alter the mechanical properties and permeability of ECs (131, 132). ECs barrier protection mediators, such as prostaglandin (PG) E2, sphingosine 1-phosphate and PGI2, reduce EC stiffness and permeability (131, 133). AECAs bind to specific antigens on the ECs and support to the EC release of mediators such as ICAM-1 and TNF-α which increase EC stiffness and permeability, leading to ECs barrier impairment (126, 134).

**Figure 4:**
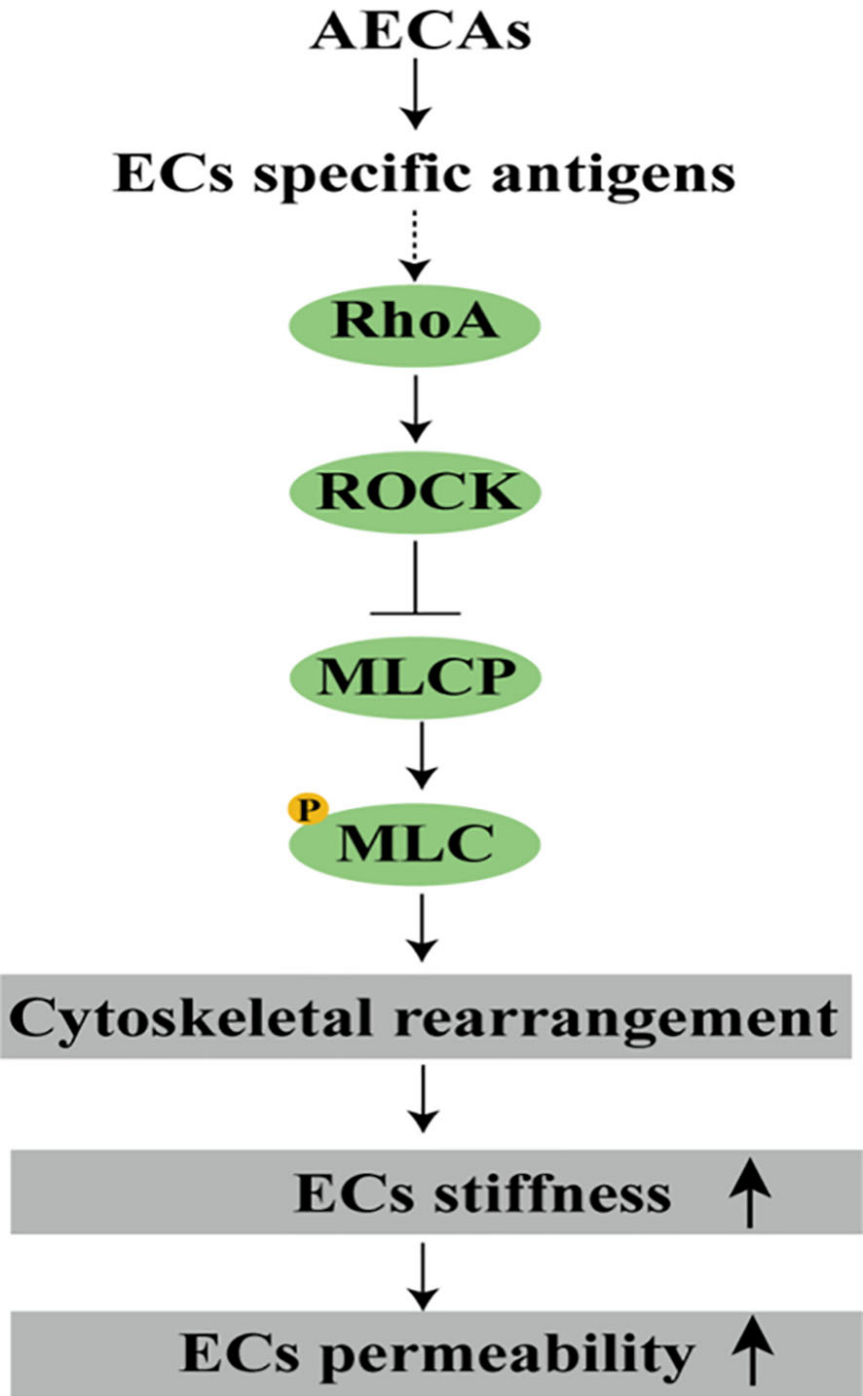
AECAs alter ECs mechanics properties. AECAs bind to specific antigens on ECs, and upon activation of the RhoA signaling pathway, ROCK is activated, inhibiting MLCP and inducing p-MLC, leading to cytoskeletal reorganization, increased ECs stiffness, and increased permeability (ROCK, Rho-related kinase; MLCP, myosin light chain phosphatase; p-MLC, MLC phosphorylation).

Pro-inflammatory cytokines stimulate or inhibit the formation of actin cytoskeletal structures. EC stiffness increases when affected by TNF-α (135). The RhoA pathway regulates TNF-α-induced cytoskeleton rearrangement, leading to increased ECs permeability (134, 136). The RhoA/ROCK pathway can regulate vascular function because changes in the actin cytoskeleton are fundamental to vascular contraction and remodeling, inflammatory cell recruitment, and cell proliferation (137). RhoA has been extensively studied as a regulator of vascular leakage and leukocyte migration through the endothelium. RhoA signals through the activation of ROCK, which inhibits MLC phosphatase and induces the phosphorylation of MLC (p-MLC) (138, 139). This process enhances the formation of actin bundles, fibers, and stretched actin structures and promotes the loss of VE-cadherin mediated cell-cell contacts, leading to vascular leakage (140–142). However, whether AECAs activate the RhoA signaling pathway by binding to antigens on the ECs’ surface and producing inflammatory factors, which leads to cytoskeletal rearrangement and increased cell stiffness and permeability, ultimately resulting in the development of vasculitis, has not been investigated and requires follow-up experiments for verification (**Figure 4**).

Despite the types of AECAs that are already known today (143, 144), there is little information about their specific antigens in ECs. Only a few antigens targeted by AECAs were identified in vasculitis (38). Among these antigens, some antigens are directly related to the cytoskeleton, such as tropomyosin and vimentin. Literature data show that tropomyosin regulates cell stiffness in a complex way via the generation of specific populations of actin filaments containing tropomyosin isoforms (145). Vimentin maintains cell mechanical properties, motility, adhesion, and other signaling pathways that protect against compressive stress and preserve mechanical integrity by enhancing cell elastic behavior (146). Several antigens, like peroxiredoxins and MPO, affect cytoskeletal activity. MPO induces actin cytoskeleton reorganization and affects mechanical stiffness, as found in human platelets (147). Peroxiredoxins interact with collapsing response mediator protein 2 that regulates microtubule structure, for example, during lymphocyte migration and neuronal development (148). Other molecular targets, like HSP60 are associated with mechanisms for regulating the functions and pathways of cell death, such as apoptosis, resulting in decreased arterial elasticity (69, 149, 150). A recent AFM study highlighted the vital role of microtubules in shaping ECs mechanics. It showed that the disruption of microtubules by exposing the cells to colchicine caused the cell to stiffen, the relaxation times increased, and the adhesion between the tip and cell decreased (151). Actin and vimentin cytoskeleton reorganization and cell stiffening have been recently detected in ECs after blocking CD109 antigen, a regulator of many signaling pathways, using anti-CD109 antibodies (152).

## Conclusion

AECAs target diverse antigenic epitopes, including planted antigens, constitutively expressed surface molecules, and cryptic antigens exposed during cellular stress. These interactions can induce ECs injury through ADCC mechanisms. Additionally, AECAs exhibit pro-angiogenic properties and may alter ECs mechanical properties, such as cytoskeletal integrity and intercellular junction stability, thereby promoting vascular hyperpermeability. Collectively, these pathophysiological processes contribute to the pathogenesis of vasculitis. However, AECAs exhibit considerable heterogeneity in their prevalence across vasculitis, and the correlation between antibody titers and clinical disease activity remains inconsistent across studies. Current limitations in their clinical utility stem from suboptimal specificity and the absence of standardized detection protocols. Therefore, methodologically rigorous studies are required to establish reproducible assay systems for AECAs quantification. Such standardization is critical to elucidate the precise mechanistic roles of AECAs in vasculitis progression and to evaluate their potential as therapeutic targets or disease-monitoring biomarkers.
